# A new species of *Plutella* Schrank (Lepidoptera, Plutellidae) from the Andes of northern Chile

**DOI:** 10.3897/BDJ.12.e139664

**Published:** 2024-11-26

**Authors:** Héctor A. Vargas

**Affiliations:** 1 Universidad de Tarapacá, Facultad de Ciencias Agronómicas, Departamento de Recursos Ambientales, Arica, Chile Universidad de Tarapacá, Facultad de Ciencias Agronómicas, Departamento de Recursos Ambientales Arica Chile

**Keywords:** arid environments, diamondback moth, Neotropical Region, taxonomy, South America

## Abstract

**Background:**

The moth genus *Plutella* Schrank, 1802 (Lepidoptera, Plutellidae) includes 26 described species. In addition to the diamondback moth, *P.xylostella* (Linnaeus, 1758), which is an important and widely distributed pest of cruciferous crops, only two species have been previously recorded in Chile, both with distribution ranges restricted to the southern temperate rainforests.

**New information:**

*Plutellacopaquillaensis* sp. nov. is described and illustrated, based on adults reared from larvae collected on *Neuontobotryslanata* (Walp.) Al-Shehbaz (Brassicaceae) in the arid highlands of the Andes of northern Chile. The new species resembles *P.xylostella* in wing pattern, but clearly differs in genitalia morphology.

## Introduction

*Plutella* Schrank, 1802 (Lepidoptera, Plutellidae) is a widespread moth genus that currently includes 26 described species (Robinson and Sattler 2001, Baraniak 2007, Landry and Hebert 2013, Søli et al. 2018, Sohn 2023). Like many members of Plutellidae, larvae of *Plutella* primarily feed on plants in the family Brassicaceae (Robinson and Sattler 2001, Heckford and Beavan 2011, Abram et al. 2022), with the exception of lineages endemic to the Hawaiian Islands, whose documented host plants belong to the family Capparaceae (Swezey 1920, Robinson and Sattler 2001), a member of the order Brassicales closely related to Brassicaceae and Cleomaceae (Su et al. 2012). The delimitation of *Plutella* is controversial due to the wide variation in genitalia morphology amongst the currently included species (Robinson and Sattler 2001, Baraniak 2007, Landry and Hebert 2013, Sohn 2023). Based on the study of the Palearctic fauna, Baraniak (2007) restricted the genus to its type species and one close relative (*P.karsholtella* Baraniak, 2007) that was later synonymised with *P.xylostella* (Landry and Hebert 2013) and described two other genera that were later synonymised with *Plutella* (Sohn 2023).

The best-known representative of *Plutella* is its type species *P.xylostella* (Linnaeus, 1758), the diamondback moth, a widely distributed and economically important pest of cruciferous crops (Robinson and Sattler 2001, Zalucki et al. 2012, Furlong et al. 2013). This species represented a morphologically distinct member of the genus (Robinson and Sattler 2001) until the discovery of *P.australiana* Landry & Hebert, 2013 from eastern Australia, whose genitalia morphology and mitochondrial DNA divergence suggest its closeness to *P.xylostella* (Landry and Hebert 2013). This discovery highlights the importance that progress in knowledge of taxonomic diversity can have in improving the delimitation of *Plutella*. Another aspect that remained uncertain for a long time was the geographic origin of the diamondback moth (Robinson and Sattler 2001). Although an Old World origin was previously hypothesised (Kfir 1998), the results of a recent genomic study, based on a global sample collection, indicated that this species would have originated in South America (You et al. 2020). Thus, the diamondback moth would have evolved associated with Brassicales hosts native to this area and shifted to use cruciferous crops after the arrival of Europeans (You et al. 2020).

Unlike the large number of studies dealing with *P.xylostella*, many members of this genus remain poorly known (Heckford and Beavan 2011, Søli et al. 2018), including those described from South America (Robinson and Sattler 2001). In addition to the diamondback moth, only two *Plutella* species have been previously recorded in Chile, *P.deltodoma* Meyrick, 1931 and *P.diluta* Meyrick, 1931, both with distribution ranges restricted to the southern temperate rainforests (Meyrick 1931, Wakeham-Dawson 2022). Rearing of larvae collected on a cruciferous plant native to the arid Andes of northern Chile allowed me to obtain *Plutella* moths whose wing pattern closely resembles that of *P.xylostella*. Subsequent examination of the genitalia morphology revealed that the obtained moths differed enough to represent a new species whose taxonomic description is provided here.

## Materials and methods

Larvae were collected on *Neuontobotryslanata* (Walp.) Al-Shehbaz (Brassicaceae) in May 2022 in the Copaquilla Ravine (18°24'02"S, 69°38'37"W) at about 3100 m elevation on the western slope of the Andes in the Parinacota Province of northern Chile. Adults emerged in June 2022. The abdomen of each adult was removed and placed in hot potassium hydroxide (KOH) 10% for a few minutes for dissection of the genitalia, which were stained with Eosin Y and Chlorazol Black and mounted on slides with Euparal. Photos were taken with an iPhone 11 camera attached to a Leica M125 stereomicroscope and a Leica MC170 HD camera attached to a Leica DM1000 LED light microscope. The holotype, paratypes and their genitalia slides are deposited in the “Colección Entomológica de la Universidad de Tarapacá” (IDEA), Arica, Chile.

## Taxon treatments

### Plutella copaquillaensis

Vargas sp. nov.

AAA6D32E-C771-501C-9867-AEAB5178DA8E

B53949BF-378E-427F-BE6F-EF68D02814D8

#### Materials

- **Type status:** Holotype. **Occurrence:** catalogNumber: IDEA-LEPI-2024-14; sex: male; preparations: Genitalia slide HAV-1814; occurrenceID: C050BB01-35C7-5A54-8CEE-856B03AF7D00; **Location:** country: Chile; stateProvince: Parinacota; locality: Copaquilla; verbatimElevation: 3100 m; verbatimLatitude: 18°24'02"S; verbatimLongitude: 69°38'37"W; **Identification:** identifiedBy: Héctor A. Vargas; **Event:** samplingProtocol: Adult emerged June 2022, reared from larva collected on Neontobotrys lanata May 2022; **Record Level:** type: PhysicalObject; language: en; institutionCode: IDEA (Colección Entomológica de la Univerdidad de Tarapacá); basisOfRecord: PreservedSpecimen
- **Type status:** Paratype. **Occurrence:** catalogNumber: IDEA-LEPI-2024-15; sex: male; preparations: Genitalia slide HAV-1759; occurrenceID: 26D3FF6E-6D65-51DF-AD5E-93A7B7556864; **Location:** country: Chile; stateProvince: Parinacota; locality: Copaquilla; verbatimElevation: 3100 m; verbatimLatitude: 18°24'02"S; verbatimLongitude: 69°38'37"W; **Identification:** identifiedBy: Héctor A. Vargas; **Event:** samplingProtocol: Adult emerged June 2022, reared from larva collected on Neontobotrys lanata May 2022; **Record Level:** type: PhysicalObject; language: en; institutionCode: IDEA (Colección Entomológica de la Univerdidad de Tarapacá); basisOfRecord: PreservedSpecimen
- **Type status:** Paratype. **Occurrence:** catalogNumber: IDEA-LEPI-2024-16; sex: male; preparations: Genitalia slide HAV-1761; occurrenceID: 26D3FF6E-6D65-51DF-AD5E-93A7B7556864; **Location:** country: Chile; stateProvince: Parinacota; locality: Copaquilla; verbatimElevation: 3100 m; verbatimLatitude: 18°24'02"S; verbatimLongitude: 69°38'37"W; **Identification:** identifiedBy: Héctor A. Vargas; **Event:** samplingProtocol: Adult emerged June 2022, reared from larva collected on Neontobotrys lanata May 2022; **Record Level:** type: PhysicalObject; language: en; institutionCode: IDEA (Colección Entomológica de la Univerdidad de Tarapacá); basisOfRecord: PreservedSpecimen
- **Type status:** Paratype. **Occurrence:** catalogNumber: IDEA-LEPI-2024-17; sex: male; preparations: Genitalia slide HAV-1765; occurrenceID: 709C1C9B-FA97-50EB-8739-543CB9B6DE11; **Location:** country: Chile; stateProvince: Parinacota; locality: Copaquilla; verbatimElevation: 3100 m; verbatimLatitude: 18°24'02"S; verbatimLongitude: 69°38'37"W; **Identification:** identifiedBy: Héctor A. Vargas; **Event:** samplingProtocol: Adult emerged June 2022, reared from larva collected on Neontobotrys lanata May 2022; **Record Level:** type: PhysicalObject; language: en; institutionCode: IDEA (Colección Entomológica de la Univerdidad de Tarapacá); basisOfRecord: PreservedSpecimen
- **Type status:** Paratype. **Occurrence:** catalogNumber: IDEA-LEPI-2024-18; sex: male; preparations: Genitalia slide HAV-1767; occurrenceID: 76479FD3-49BB-5CF5-A9F6-98EA891F5FE6; **Location:** country: Chile; stateProvince: Parinacota; locality: Copaquilla; verbatimElevation: 3100 m; verbatimLatitude: 18°24'02"S; verbatimLongitude: 69°38'37"W; **Identification:** identifiedBy: Héctor A. Vargas; **Event:** samplingProtocol: Adult emerged June 2022, reared from larva collected on Neontobotrys lanata May 2022; **Record Level:** type: PhysicalObject; language: en; institutionCode: IDEA (Colección Entomológica de la Univerdidad de Tarapacá); basisOfRecord: PreservedSpecimen
- **Type status:** Paratype. **Occurrence:** catalogNumber: IDEA-LEPI-2024-19; sex: male; preparations: Genitalia slide HAV-1768; occurrenceID: 588A74E8-4860-549B-B8FD-DA742F1D3D67; **Location:** country: Chile; stateProvince: Parinacota; locality: Copaquilla; verbatimElevation: 3100 m; verbatimLatitude: 18°24'02"S; verbatimLongitude: 69°38'37"W; **Identification:** identifiedBy: Héctor A. Vargas; **Event:** samplingProtocol: Adult emerged June 2022, reared from larva collected on Neontobotrys lanata May 2022; **Record Level:** type: PhysicalObject; language: en; institutionCode: IDEA (Colección Entomológica de la Univerdidad de Tarapacá); basisOfRecord: PreservedSpecimen
- **Type status:** Paratype. **Occurrence:** catalogNumber: IDEA-LEPI-2024-20; sex: female; preparations: Genitalia slide HAV-1541; occurrenceID: 45B9A8C2-7707-54B6-8DDE-39FEAC988191; **Location:** country: Chile; stateProvince: Parinacota; locality: Copaquilla; verbatimElevation: 3100 m; verbatimLatitude: 18°24'02"S; verbatimLongitude: 69°38'37"W; **Identification:** identifiedBy: Héctor A. Vargas; **Event:** samplingProtocol: Adult emerged June 2022, reared from larva collected on Neontobotrys lanata May 2022; **Record Level:** type: PhysicalObject; language: en; institutionCode: IDEA (Colección Entomológica de la Univerdidad de Tarapacá); basisOfRecord: PreservedSpecimen
- **Type status:** Paratype. **Occurrence:** catalogNumber: IDEA-LEPI-2024-21; sex: female; preparations: Genitalia slide HAV-1760; occurrenceID: 513968D5-BFA6-5375-844A-935F7038A2D0; **Location:** country: Chile; stateProvince: Parinacota; locality: Copaquilla; verbatimElevation: 3100 m; verbatimLatitude: 18°24'02"S; verbatimLongitude: 69°38'37"W; **Identification:** identifiedBy: Héctor A. Vargas; **Event:** samplingProtocol: Adult emerged June 2022, reared from larva collected on Neontobotrys lanata May 2022; **Record Level:** type: PhysicalObject; language: en; institutionCode: IDEA (Colección Entomológica de la Univerdidad de Tarapacá); basisOfRecord: PreservedSpecimen
- **Type status:** Paratype. **Occurrence:** catalogNumber: IDEA-LEPI-2024-22; sex: female; preparations: Genitalia slide HAV-1764; occurrenceID: 02A03212-7AAC-50F3-9B3E-9C3ADF5CA687; **Location:** country: Chile; stateProvince: Parinacota; locality: Copaquilla; verbatimElevation: 3100 m; verbatimLatitude: 18°24'02"S; verbatimLongitude: 69°38'37"W; **Identification:** identifiedBy: Héctor A. Vargas; **Event:** samplingProtocol: Adult emerged June 2022, reared from larva collected on Neontobotrys lanata May 2022; **Record Level:** type: PhysicalObject; language: en; institutionCode: IDEA (Colección Entomológica de la Univerdidad de Tarapacá); basisOfRecord: PreservedSpecimen
- **Type status:** Paratype. **Occurrence:** catalogNumber: IDEA-LEPI-2024-23; sex: female; preparations: Genitalia slide HAV-1770; occurrenceID: 425DDB72-797B-59AC-AFD1-1B592C14622F; **Location:** country: Chile; stateProvince: Parinacota; locality: Copaquilla; verbatimElevation: 3100 m; verbatimLatitude: 18°24'02"S; verbatimLongitude: 69°38'37"W; **Identification:** identifiedBy: Héctor A. Vargas; **Event:** samplingProtocol: Adult emerged June 2022, reared from larva collected on Neontobotrys lanata May 2022; **Record Level:** type: PhysicalObject; language: en; institutionCode: IDEA (Colección Entomológica de la Univerdidad de Tarapacá); basisOfRecord: PreservedSpecimen
- **Type status:** Paratype. **Occurrence:** catalogNumber: IDEA-LEPI-2024-24; sex: female; preparations: Genitalia slide HAV-1815; occurrenceID: 3585D1A3-ADBE-5956-B668-63498E14B48F; **Location:** country: Chile; stateProvince: Parinacota; locality: Copaquilla; verbatimElevation: 3100 m; verbatimLatitude: 18°24'02"S; verbatimLongitude: 69°38'37"W; **Identification:** identifiedBy: Héctor A. Vargas; **Event:** samplingProtocol: Adult emerged June 2022, reared from larva collected on Neontobotrys lanata May 2022; **Record Level:** type: PhysicalObject; language: en; institutionCode: IDEA (Colección Entomológica de la Univerdidad de Tarapacá); basisOfRecord: PreservedSpecimen

#### Description

**Figure 1.**
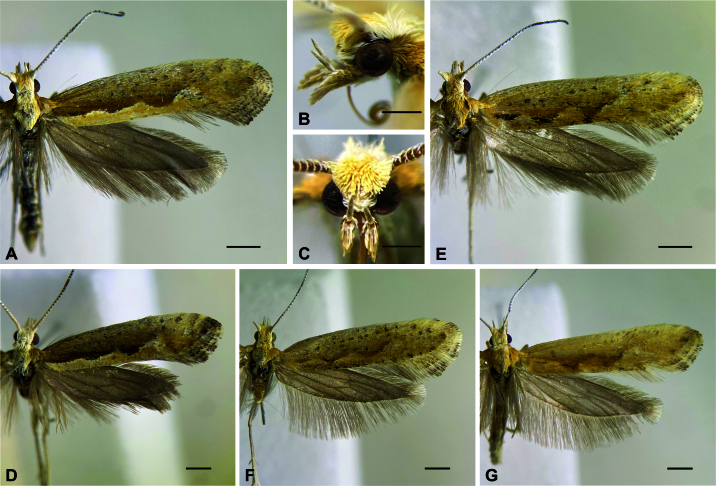
Habitus of *Plutellacopaquillaensis* sp. nov. **A** Holotype, dorsal; **B** Holotype head, lateral; **C** Holotype head, anterior; **D** Paratype male, dorsal; **E** Paratype female, dorsal; **F** Paratype female, dorsal; **G** Paratype female, dorsal. Scale bars 1, 0.5, 0.5, 1, 1, 1, 1 mm, respectively.

**Figure 2.**
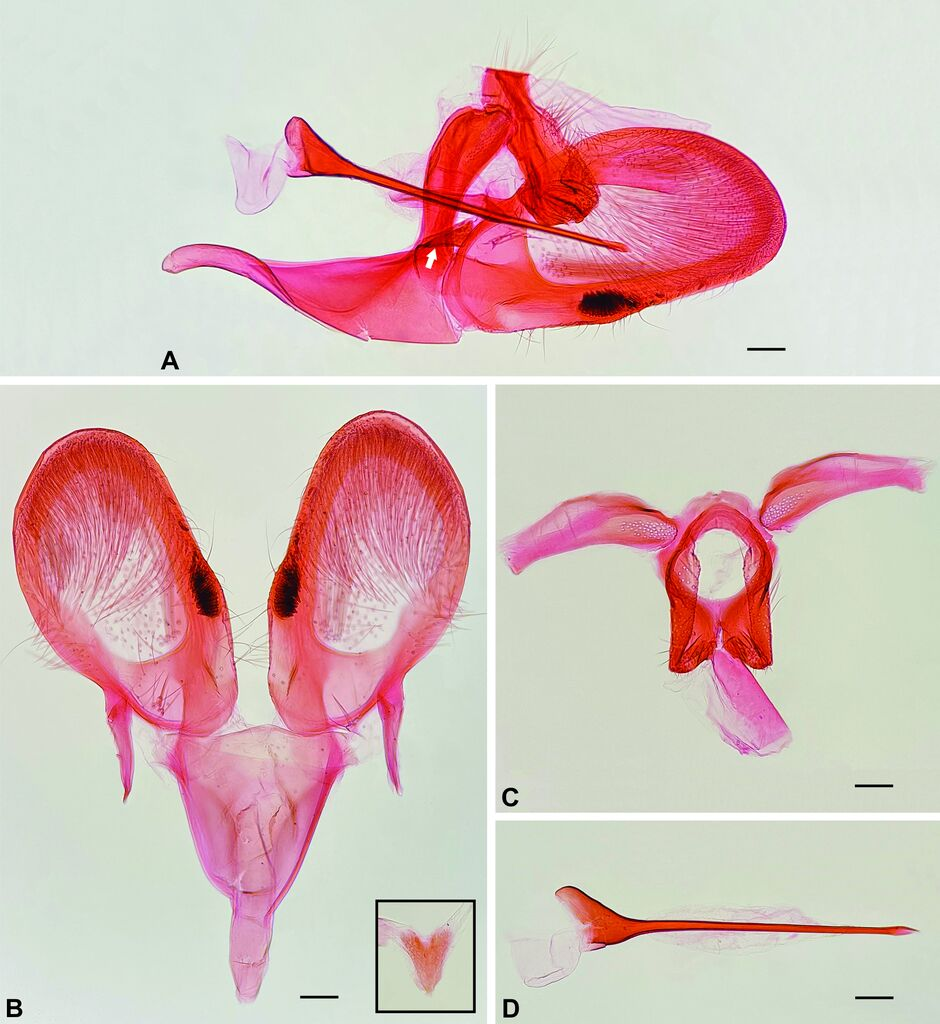
Male genitalia of *Plutellacopaquillaensis* sp. nov. **A** Male genitalia, lateral, left valva removed; white arrow indicates juxta; **B** Saccus and valvae, unrolled, ventral; bottom right rectangle: juxta, ventral; **C** Tegumen and anal cone, unrolled, dorsal; **D** Phallus, lateral. Scale bars 0.1 mm.

**Male** (Fig. 1). Forewing length 7.1–7.5 mm. **Head.** Vertex mostly pale yellow, creamy white behind antenna; frons creamy white; occiput creamy white behind vertex, yellowish-brown behind eye; ocellus present. Labial palpus porrect; first segment creamy white; second segment triangular in lateral view due to forward projected scales, outer surface yellowish-brown, inner surface creamy white; third segment upturned, mostly creamy white with scattered yellowish-brown scales. Antenna with scape and pedicel creamy white, flagellum creamy white dorsally and single transverse stripe of creamy white scales ventrally on each flagellomere. **Thorax**. Mostly creamy white dorsally, with scattered pale yellow scales; tegula yellowish-brown. Foreleg mostly brownish-grey with scattered creamy white scales; mid-leg mostly creamy white with scattered brownish-grey scales; hind leg creamy white. Forewing upper surface mostly yellowish-brown with scattered creamy white, pale yellow and blackish-brown scales on basal three quarters; dorsal band extended from base to about three quarters of forewing length, pale yellow, anterior margin with three scallops, the outer expanded by a patch of creamy white scales; a yellowish-brown band with poorly-defined anterior margin lines the dorsal band from base to just before the third scallop; apical area mostly pale yellow with scattered yellowish-brown and blackish-brown scales; lower surface brownish-grey; fringe with narrow creamy white scales with blackish-brown or yellowish-brown tip. Some specimens with a blackish-brown, instead of yellowish-brown, band lining the dorsal band. Hind wing upper and lower surfaces and fringe brownish-grey. **Abdomen**. Mostly brownish-grey with scattered creamy white scales. Segment VIII with well-developed pleural lobes and pair of coremata. **Male genitalia** (Fig. 2). Uncus absent. Tegumen a narrow, sclerotised transverse band; with pair of ventral pedunculi, each broadly fused to the respective arm of the vinculum; with pair of teguminal processes slightly longer than pedunculi, widely separated dorsally, ventrally fused near the middle. Anal cone mostly membranous, with slightly sclerotised ventral longitudinal patch slightly shorter than teguminal processes. Gnathos absent. Saccus continuous with vinculum, about three times the length of the ventral pedunculi of the tegumen; posterior two-thirds a triangular, concave plate, anterior third finger-like. Juxta triangular, about 0.25x length of saccus. Valva semicircular, about 1.2x length of saccus, maximum height about half length; ventral margin nearly straight, slightly convex near middle; apex and dorsal margin rounded; inner wall with sacculus reaching the convex part of the ventral margin of the valva, with a cluster of spiniform setae in distal area; outer wall with broad membranous area with outline similar to that of valva. Phallus slightly longer than valva, mostly needle-like, with bulbous base and pointed tip, vesica without cornuti; bulbus ejaculatorius semicircular, similar in size to base of phallus.

**Figure 3.**
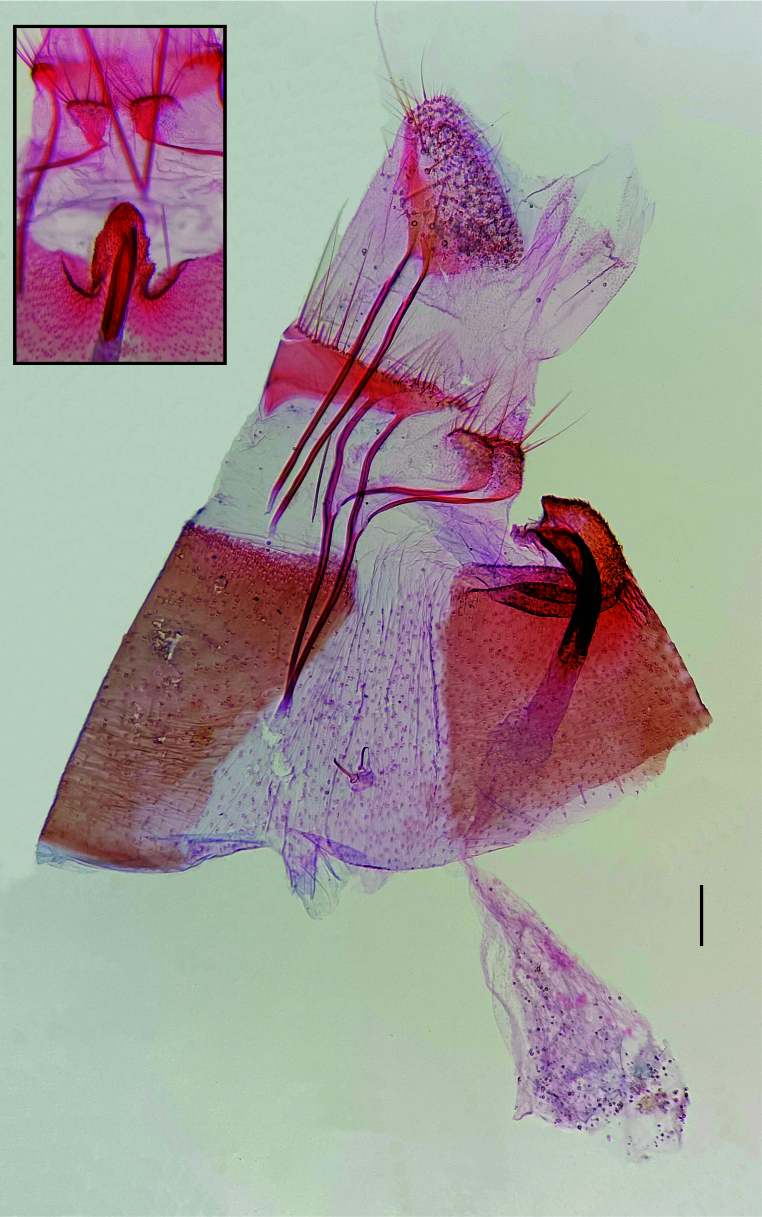
Female genitalia of *Plutellacopaquillaensis* sp. nov., lateral view; upper left rectangle: sterigma, ventral view. Scale bar 0.1 mm.

**Female** (Fig. 1). Forewing length 7.1–7.6 mm. Similar to male, but head and thorax more yellowish-brown and lighter and more variable forewing pattern, with dorsal band of forewing poorly differentiated. **Female genitalia** (Fig. 3). Papillae anales slightly sclerotised, with scattered setae. Posterior apophyses straight, about 1.7x length of papillae anales. Tergum VIII a transverse stripe with small triangular expansion in the middle of anterior margin and setae on posterior margin. Anterior apophyses slightly longer than posterior apophyses, mostly straight, slightly curved near base, with ventral branch extended to the anterior tip of the respective part of the lamella postvaginalis. Lamella postvaginalis a pair of triangular sclerites with setae on posterior margin. Sternum VII with slightly upcurved posterior projection arising from the middle of the posterior margin between two small excavations; length about 1.7x basal width. Ostium bursae at the tip of the posterior projection of sternum VII. Ductus bursae with well-sclerotised, upcurved posterior half, about twice the length of posterior projection of sternum VII; anterior half membranous, straight. Corpus bursae elongated, pear-shaped, membranous, without signa.

#### Diagnosis

**Figure 4.**
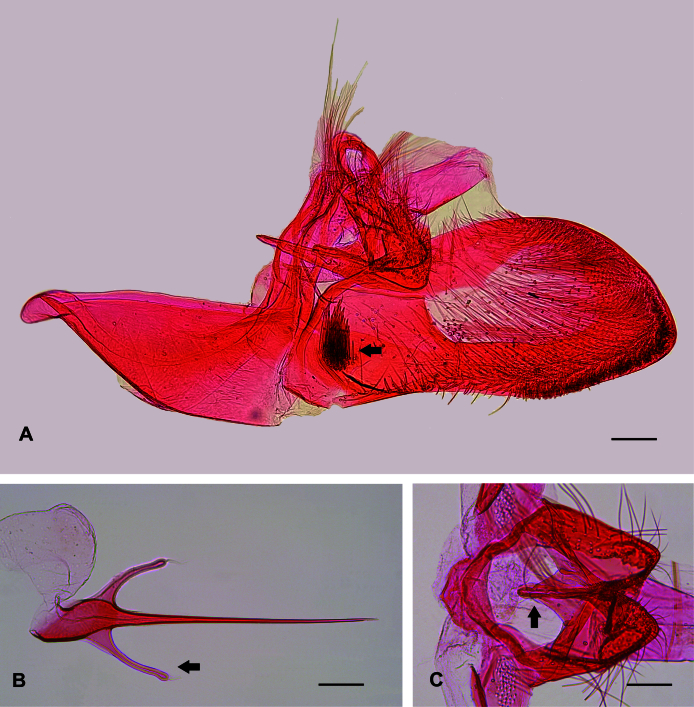
Male genitalia of *Plutellaxylostella* (Linnaeus, 1758) showing differences with *Plutellacopaquillaensis* sp. nov. **A** Male genitalia, lateral, left valva and phallus removed; black arrow indicates cluster of spiniform setae in the sacculus near the base of the valva; **B** Phallus, dorsal; black arrow indicates one of the two basal hook-like processes; **C** Tegumen, gnathos and anal cone, dorsal; black arrow indicates gnathos. Scale bars 0.1, 0.1, 0.12 mm, respectively.

**Figure 5.**
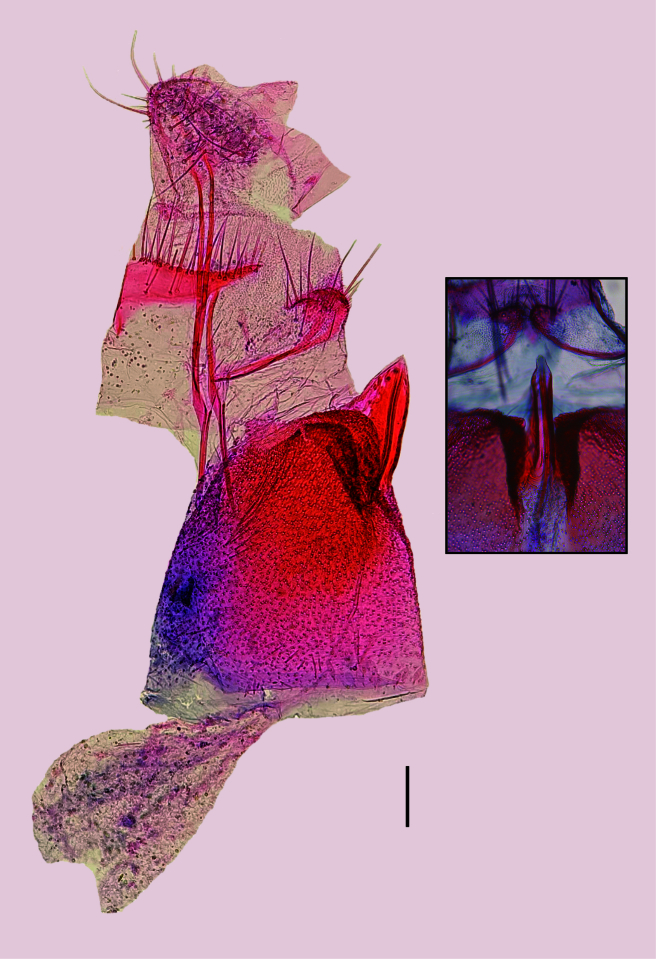
Female genitalia of *Plutellaxylostella* (Linnaeus, 1758) showing differences with *Plutellacopaquillaensis* sp. nov., lateral view; right rectangle: sterigma, ventral view. Scale bar 0.1 mm.

**Figure 6.**
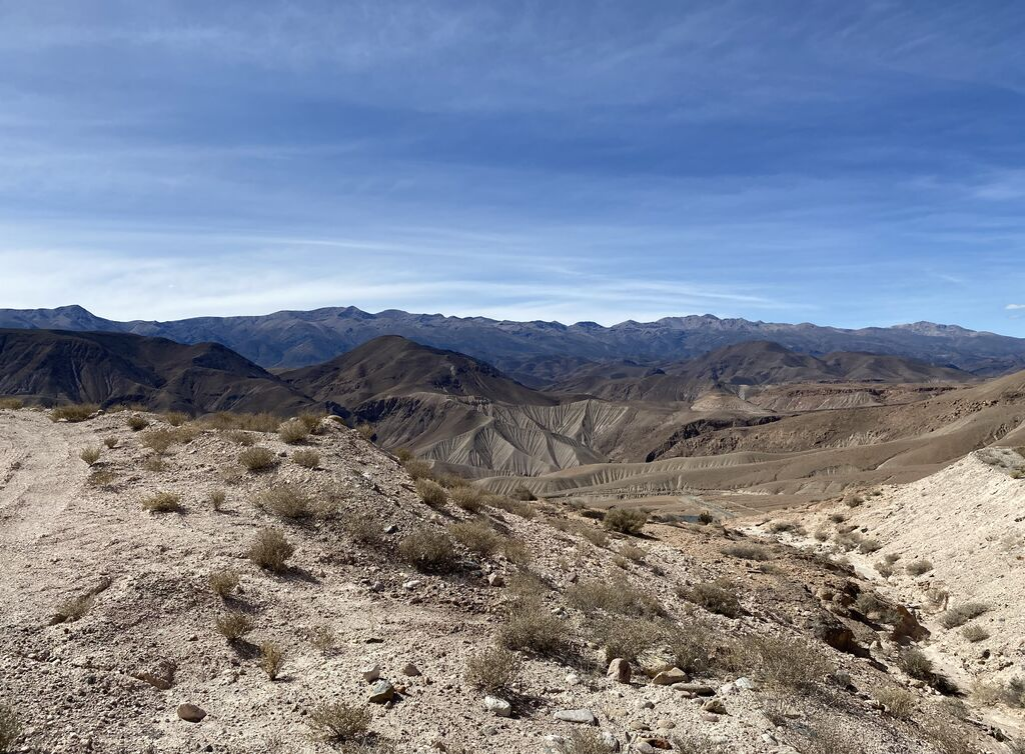
Type locality of *Plutellacopaquillaensis* sp. nov., the Copaquilla Ravine, at about 3100 m elevation on the western slope of the Andes of northern Chile.

**Figure 7.**
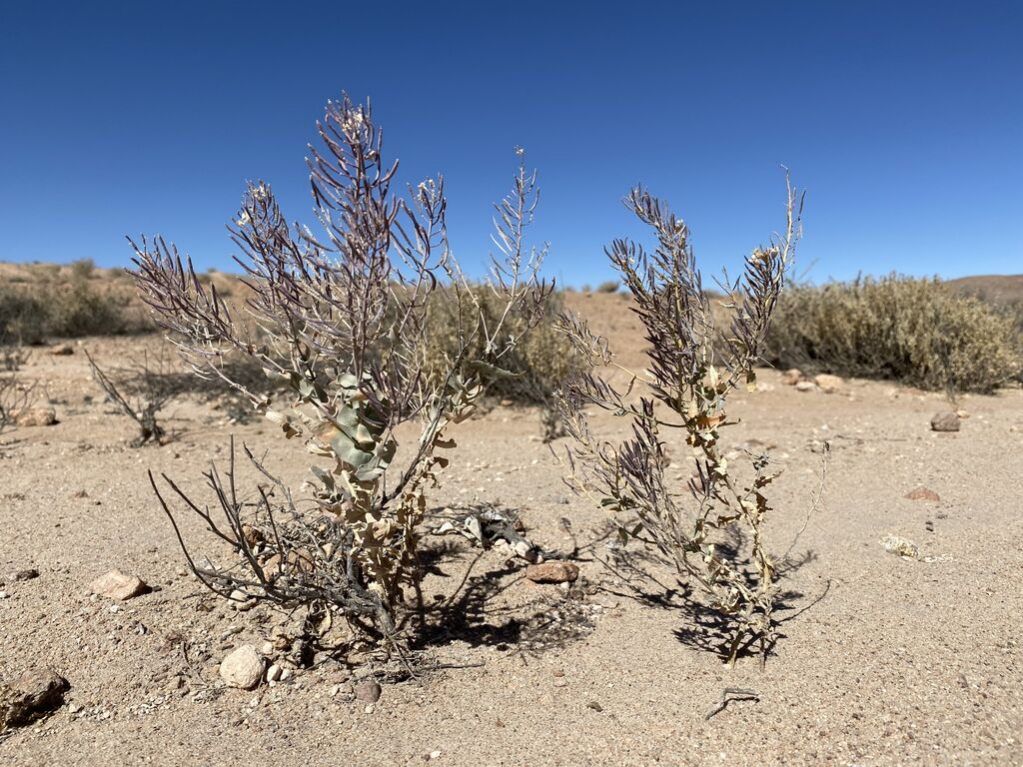
*Neuontobotryslanata* (Walp.) Al-Shehbaz (Brassicaceae), the host plant of *Plutellacopaquillaensis* sp. nov. in the Andes of northern Chile.

*Plutellacopaquillaensis* sp. nov. is recognised. based on genitalia morphology. The male lacks a gnathos and basal hook-like processes of the phallus ("lateral hooks" of Baraniak (2007)), has a triangular juxta and a cluster of spiniform setae on the sacculus near the convex part of the ventral margin of the valva and the female has the posterior projection on sternum VII arising between two small excavations. The forewing pattern of *P.copaquillaensis* sp. nov. is variable and closely resembles that of *P.xylostella* (Landry and Hebert 2013; figs. 10–16). Although the forewing of some specimens of *P.copaquillaensis* sp. nov. is more yellowish than that of *P.xylostella*, much of the variation overlaps between the two species. However, differences in genitalia morphology allow accurate identification. Unlike *P.copaquillaensis* sp. nov., *P.xylostella* has a gnathos and a pair of basal hook-like processes on the base of the phallus, lacks a juxta and has a cluster of spiniform setae on the sacculus near the base of the valva and offset from the margin in the male (Fig. 4) and the posterior projection on sternum VII arises between two markedly raised folds in the female (Fig. 5). The forewing pattern of *P.copaquillaensis* sp. nov. also closely resembles that of *P.australiana* (Landry and Hebert 2013; figs. 3–9). However, the two species can be separated using the same characteristics of the male and female genitalia that allow the separation of *P.xylostella*.

#### Etymology

The specific epithet is derived from the type locality.

#### Distribution

*Plutellacopaquillaensis* sp. nov. is known only from the type locality, the Copaquilla Ravine, at about 3100 m elevation on the western slope of the Andes of northern Chile (Fig. 6).

#### Biology

*Neuontobotryslanata* (Walp.) Al-Shehbaz (Brassicaceae) is the only host plant currently documented for *P.copaquillaensis* sp. nov. (Fig. 7). The distribution range of *N.lanata* extends from southern Peru to central Chile (Al-Shehbaz 2006). Larvae of *P.copaquillaensis* sp. nov. feed on the flowers of this plant.

## Discussion

The morphological delimitation of *Plutella* and related genera remains controversial (Sohn 2023). In the meantime, progress in the knowledge of the taxonomic diversity of Plutellidae may provide useful information for planning future studies that improve the understanding of the phylogenetic relationships and stabilise the delimitation of these genera (Robinson and Sattler 2001, Baraniak 2007, Landry and Hebert 2013, Huemer and Sohn 2020).

In addition to the remarkable similarity of the wing pattern of *P.copaquillaensis* sp. nov. to that of *P.xylostella* and *P.australiana*, the new species shows some interesting similarities with this pair of species in genitalia morphology: a cluster of spiniform setae on the sacculus of the male and sternum VII with a posterior projection arising from the middle of the posterior margin in the female. However, at least three attributes in the male genitalia of the new species suggest that it is distantly related to *P.xylostella* and *P.australiana*: absence of gnathos, absence of a pair of basal hook-like processes of the phallus and the presence of juxta. Considering the current circumscription of *Plutella* (Robinson and Sattler 2001, Landry and Hebert 2013, Søli et al. 2018, Sohn 2023), it is difficult to suggest that any member of the genus is closely related to *P.copaquillaensis* sp. nov. Further studies would be needed to reveal the phylogenetic relationships of this species.

*Plutellacopaquillaensis* sp. nov. clearly differs from the two members of the genus previously recorded in Chile. *Plutelladeltodoma* and *P.diluta* have a narrow saccus and lack a cluster of spiniform setae on the sacculus of the male genitalia (Clarke 1965; plate 184, figs. 3b and 4b, respectively).

Members of Plutellidae are mostly oligophagous leaf webbers associated with Brassicales (Sohn et al. 2013). The use of a host plant belonging to the family Brassicaceae by *P.copaquillaensis* sp. nov. fits the more commonly recorded pattern for members of *Plutella* (Robinson and Sattler 2001, Heckford and Beavan 2011, Abram et al. 2022). Although larvae of *Plutella* mostly feed on leaves (Robinson and Sattler 2001, Heckford and Beavan 2011), those of *P.copaquillaensis* sp. nov. mostly feed on flowers, like those of the North American *P.armoraciae* Busck, 1912 (Abram et al. 2022). Further studies should explore the host plant range of the new species to assess whether it only uses native hosts or can also feed on cultivated cruciferous. Furthermore, as detailed knowledge of the natural history of non-pest *Plutella* species can be useful to improve control practices of the diamondback moth (Abram et al. 2022), additional studies addressing the biology and natural enemies of *P.copaquillaensis* sp. nov. could yield valuable results for developing a more sustainable management of *P.xylostella* in the arid environments of northern Chile.

## Supplementary Material

XML Treatment for Plutella copaquillaensis
